# High Density Sphere Culture of Adult Cardiac Cells Increases the Levels of Cardiac and Progenitor Markers and Shows Signs of Vasculogenesis

**DOI:** 10.1155/2013/696837

**Published:** 2012-12-27

**Authors:** Kristina Vukusic, Marianne Jonsson, Camilla Brantsing, Göran Dellgren, Anders Jeppsson, Anders Lindahl, Julia Asp

**Affiliations:** ^1^Department of Clinical Chemistry and Transfusion Medicine, Institute of Biomedicine, the Sahlgrenska Academy, University of Gothenburg, 413 45 Gothenburg, Sweden; ^2^Department of Molecular and Clinical Medicine, the Sahlgrenska Academy, University of Gothenburg, 413 45 Gothenburg, Sweden; ^3^Department of Cardiothoracic Surgery, Sahlgrenska University Hospital, 413 45 Gothenburg, Sweden

## Abstract

3D environment and high cell density play an important role in restoring and supporting the phenotypes of cells represented in cardiac tissues. The aim of this study was therefore to investigate the suitability of high density sphere (HDS) cultures for studies of cardiomyocyte-, endothelial-, and stem-cell biology. Primary adult cardiac cells from nine human biopsies were cultured using different media for up to 9 weeks. The possibilities to favor a certain cell phenotype and induce production of extra cellular matrix (ECM) were studied by histology, immunohistochemistry, and quantitative real-time PCR. Defined media gave significant increase in both cardiac- and progenitor-specific markers and also an intraluminal position of endothelial cells over time. Cardiac media showed indication of differentiation and maturity of HDS considering the ECM production and activities within *NOTCH* regulation but no additional cardiac differentiation. Endothelial media gave no positive effects on endothelial phenotype but increased proliferation without fibroblast overgrowth. In addition, indications for early vasculogenesis were found. It was also possible to affect the Wnt signaling in HDS by addition of a glycogen synthase kinase 3 (GSK3) inhibitor. In conclusion, these findings show the suitability of HDS as *in vitro* model for studies of cardiomyocyte-, endothelial-, and stem-cell biology.

## 1. Introduction

Adult human myocardium has extremely slow cell turnover [1], once damaged it is hardly regenerated at all. Therefore, the formation of 3D constructs with cells capable to differentiate into functional cardiomyocytes and supportive cell phenotypes in the right proportions is the goal in tissue engineering and cardiac regenerative medicine. Of particular interest is the ability to restore the cardiomyocyte, which harbors the contractile function of the heart, after myocardial infarction. Different mechanisms have been proposed for cardiac regeneration. One is recruitment of endogenous progenitor cells, either from bone marrow or stem-cell niches within the heart, to the damaged area [2, 3]. Alternatively, cardiomyocytes have been suggested to reenter the cell cycle. This has been seen in zebrafish, where a heart repairing unit consisting of *GATA4* expressing cardiomyocytes has activated proliferation [4]. Proliferation of cardiomyocytes has also been found in rodents where dedifferentiated cardiomyocytes started to express *C-KIT*, *GATA4* and *NKX2.5* and to proliferate in monolayer culture [5]. 

Regardless of mechanism for cardiomyocyte regeneration, the regenerative process is a tightly regulated cellular event. The family of Notch receptors is known to act in mesodermal differentiation processes at different stages of development [6]. It has recently been reported that the *NOTCH1* receptor is expressed by cardiac progenitor cells (CPC), and that its ligand *JAG1* is expressed by their niche supporting cells in the mouse heart. Activation of *NOTCH1* resulted in upregulation of *NKX2.5* [7]. In addition to Notch signaling, the canonical Wnt/*β*-catenin signaling pathway has been reported to play a key role in cardiac stem-cell niche regulation. There, it has been shown to be a major component of the cardiac mesenchymal cell microenvironment that modulates the prespecification, renewal, and differentiation of *ISL1+* cardiovascular progenitor cells [8]. Furthermore, it has been demonstrated that *β*-catenin signaling regulates survival and proliferation of CPC, and that its expression is required for Isl1 expression in those cells [9]. Recently, Wnt signaling was found to be negatively regulated by *NOTCH1* in CPC [10].

For studies of regenerative capacity of cardiac tissue, the behavior of cardiac cells and their regulatory pathways needs to be further elucidated. There is a need for new cell culture protocols, allowing for presence of cardiomyocytes. Adult cardiomyocytes cultured on plastic surface lose their sarcomeric structures by day one [11]. In addition, there are other challenges with monolayer cultures such as fibroblast overgrowth, cytoskeleton remodeling, and unwanted differentiation of progenitor cells. 3D models are better mimicking the *in vivo* situation; allowing for more cell-cell interactions, layer formations, architecture of extracellular matrix (ECM), and enhanced paracrine communication. Successful 3D models have been developed for many tissues (reviewed in [12]), for example, liver where bile canaliculi was formed or breast where central lumen with production of milk protein was obtained. A study on ventricular cells from rat showed a significant alteration in organization and function of cells cultured in a 3D environment compared to monolayer. This was due to cell migration and responsiveness to hormone stimulation [13].

Not many studies have been performed on cultured human adult cardiac cells due to the limited material access and perhaps limited plasticity of older cells. Cultures of cardiospheres where cells have migrated and self-organized into spheres [14–16] have shown an increase in the expression of progenitor markers such as *C-KIT*, but the cardiomyogenic potential of cells from these kind of cultures has been questioned [17]. In our previous study [18], we described a novel 3D model of high density spheres (HDS) cultured with or without cardiomyocytes. Results from that study suggested that this 3D model could harbor and nourish CPC, in which marker expression increased with culture time. On the other hand, expression of smooth muscle actin significantly decreased in culture, while expression of the endothelial cell marker *VWF* was more variable. In the present study, we analyzed HDS containing all cells isolated from the cardiac tissue. Based on our previous results, we investigated the suitability of this 3D system for studies of cardiomyocyte-, endothelial-, and stem-cell biology. For this purpose, three different culture media were used, and markers for cardiomyocytes, endothelial cells, and CPC were analyzed. Effects on proliferation, fibroblast growth, and ECM production were also studied. Moreover, the possibility to affect the Wnt/*β*-catenin signaling pathway by addition of a GSK3 inhibitor was investigated within this system. 

## 2. Material and Methods

### 2.1. Cardiac Biopsies

Cardiac tissue was obtained after informed consent from patients undergoing heart surgery and after ethical approval by the Research Ethics Board at the University of Gothenburg and the Sahlgrenska Academy, Gothenburg, Sweden. Biopsies from the auricle of right atrium were received from nine adult subjects (ages 64–84 years, 7 males, 2 females). The mean weight of the biopsies was 165 mg. Cause of operation was either coronary artery disease or heart valve disease. 

### 2.2. Isolation of Human Cardiac Cells, Culture of HDS, and Stimulation with SB216763

Heart biopsies were minced, and the pieces were digested with 0.8 mg/mL collagenase type II (Worthington, Lakewood, NJ, USA) for 20–24 h at 37°C. For each HDS, 2 × 10^5^ cells (all cell types included) were placed in a polypropylene conical tube with 0.5 mL Defined medium consisting of DMEM-high glucose (PAA Laboratories, Pasching, Austria) supplemented with 5 *μ*g/mL linoleic acid (Sigma-Aldrich, Saint Louis, MO, USA), 1% insulin-transferrin-selenium-G (ITS-G concentrate; Life Technologies, Paysley, UK), 1 mg/mL human serum albumin (Equitech-Bio, Kerrville, TX, USA), 10 ng/mL TGF-*β*1 (R&D Systems, Abingdon, UK), 10^−7^ M dexamethasone (Sigma-Aldrich), 14 *μ*g/mL ascorbic acid (Sigma-Aldrich), and 1% penicillin streptomycin (PAA Laboratories). The cells were centrifuged at 500× g for 5 min and maintained at 37°C in 5% CO_2_. HDS rounded up within a week and were floating in the medium. Media were changed twice a week. For experiments with different media, 6-week-old HDS was treated with 5-Azacytidine for 72 h and cultured in Cardiac medium [19] containing 2% human serum for 3 additional weeks. Endothelial medium (EGM-2 MV, Lonza, Basel Switzerland) was added to 1-week-old HDS, which was cultured for another 3 weeks. The GSK3 inhibitor SB216763 (Sigma-Aldrich) dissolved in DMSO was added in Defined medium to 1 week old HDSs. A final concentration of 5 *μ*M was added at media change three times during 1 week of stimulation. As control the same volume of DMSO was added to HDS in Defined medium. For gene expression analysis, HDSs were washed in phosphate-buffered saline (PBS) and frozen at −80°C until use. For histology and protein expression analysis, HDSs were fixed in Histofix (Histolab Products AB, Gothenburg, Sweden).

### 2.3. Histology

HDS were processed for histology as previously described [18] and stained with hematoxylin-eosin (HE). For detection of ECM, Alcian blue van Gieson (AbvG) and Picro Sirius Red staining were performed. Miller's elastin staining was used to visualize vascularization. Cardiac tissue sections from one of the patients were included for comparison. Nikon Optiphot2-pol light microscope (Nikon Corporation, Tokyo, Japan) was used to analyze sections.

### 2.4. Immunohistochemistry

Sections of HDS were analyzed with antibodies detecting *α*-Sarcomeric actin, *VWF* (A2172 and F3520, Sigma-Aldrich), Cardiac Troponin T, Ki67 (ab10214 and ab6526, Abcam, Cambridge, UK), *NOTCH1*, active *β*-catenin, and *JAG1* (sc-9170, sc-70511, and sc-11376, Santa Cruz Biotechnology, Santa Cruz, CA, USA). Briefly, 5 *μ*m paraffin sections were deparaffinized with xylene 2 × 10 min and rehydrated in 99, 95, and 70% ethanol for 5 min in each solution. Antigen-retrieval treatment for detection of Ki67 and active *β*-catenin was performed using 10 mM citrate buffer pH 6 for 15 min in 90°C, followed by room temperature (RT) for 30 min. All samples (except samples stained for *NOTCH1*) were treated for 15 min with 0.1% Triton X-100 and blocked with 2% bovine serum albumin/Triton X-100 in PBS for 15 min at RT. Before analysis of *NOTCH1*, samples were digested with 8000 units/mL hyaluronidase (Sigma-Aldrich) for 1 hour in 37°C and then digested with 0.05 units/mL chondroitinase AC (Sigma-Aldrich) for 1 hour in 37°C. The samples were blocked for endogenous peroxidase activity with 3% H_2_O_2_ solution for 10 min and then blocked for 15 min with 3% bovine serum albumin in PBS. All sections were incubated with primary antibodies for >16 h at 4°C and incubated for at least 1.5 h at RT with a secondary antibody conjugated to Alexa flour (Invitrogen, Carlsbad, CA, USA) or horseradish peroxidase (Millipore, Temecula, CA, USA). The antibody conjugated to horseradish peroxidase was used together with an enhancement step with TSA-CY3 Kit (Perking Elmer, Boston, MA, USA) according to manufacturer's instructions. Prolong Gold antifade containing DAPI for nuclear staining (Invitrogen) was used to cover the sections. Results were visualized by Nikon ECLIPSE 90i using ACT-1 software (Nikon Corporation). Sections of adult human heart or skin were used as positive controls. As negative control isotype controls were used instead of the primary antibody. When an isotype control was not available primary antibody was omitted (Ki67, *VWF*). 

### 2.5. RNA Isolation and Quantitative Real-Time PCR 

HDS were homogenized with a 27G-needle, and total RNA was extracted with the RNeasy Micro Kit and DNase1 in the QIAcube (Qiagen, Hilden, Germany). For cDNA synthesis and quantitative real-time PCR (qPCR) all reagents, instruments, and software were purchased from Applied Biosystems (Foster City, USA). Briefly, cDNA was prepared from total RNA using High Capacity cDNA Reverse Transcriptase Kit with random hexamers. Preamplifications were done with the TaqMan PreAmp Master Mix. qPCR analysis was performed using the instrument 7900HT. The following human TaqMan Gene Expression Assays were used: *DDR2* Hs00178815_m1,* ISL1* Hs00158126_m1, *ABCG2 *Hs00184979_m1, *MDR1* Hs00184500_m1,* C-KIT* Hs00174029_m1*, MESP1 *Hs00251489_m1, *MEF2C* Hs00231149_m1, *GATA4* Hs00171403_m1, *TBX5* Hs01052563_m1,* NKX2.5* Hs00231763_m1, *TNNT2* Hs00165960_m1, *MYH6* Hs01101425_m1, *NPPA* Hs00383231_m1,* NOTCH1 *Hs00413187_m1,* JAG1 *Hs00164982_m1,* HEY2 *Hs00232622_m1,* CD31* Hs00169777_m1, *FLK1 *Hs00176676_m1, *VWF *Hs00169795_m1, and *DKK1* Hs00183740_m1. *CREBBP *Hs00231733_m1 was used as a reference gene [18]. Samples were analyzed in duplicates, and the relative comparative method was used to analyze the qPCR data. Gene expression data are presented in relative units. Statistical significance was determined using a paired, two-sided Student's *t*-test. Logarithmic values of the gene expression data were used for statistical calculations. A value of *P* < 0.05 was considered statistically significant.

## 3. Results and Discussion

### 3.1. Study Design

**Figure 1:**
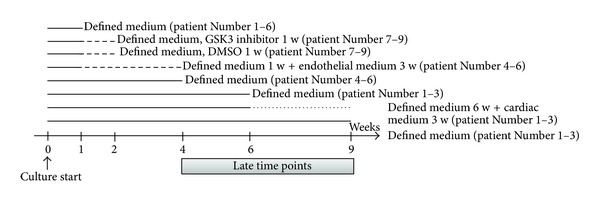
Study design. The number of biopsies representing HDS in each treatment is included in brackets.

The present work describes how a 3D culture system of HDS affects different cell phenotypes present in the native adult human heart. Since all the cells isolated from the cardiac biopsy were included in HDS in their relative proportion, we had the possibility to study the protein and gene expression of makers for several kinds of cells and the changes with time and culture condition. Based on results from our previous work [18] and the interest from a regenerative point of view, we decided to focus on CPC, cardiomyocytes and endothelial cells. HDS cultures were established from auricle of the right atrium, a location where we [18, 20] and others previously have reported detection of stem and progenitor cell markers, including *C-KIT*, Sca1, *MDR1*, and *ISL1* (reviewed in [21]). In the current experiments, HDSs were cultured for up to 9 weeks and harvested at different time points (Figure 1), where morphology and expression of markers were analyzed. Because high cell density and serum-free media restrict proliferation and allow phenotypic analysis [13], most of the phenotype analyses were performed on HDS cultured in Defined medium. To follow changes over time, 1-week-old newly formed HDS (early) was compared to 4 and 6 weeks old HDS (late). Due to the limited size of the biopsies, no characterization of dissociated cells before culture in HDS was included. Instead, characterization by FACS and qPCR of directly dissociated cells from the same group of biopsies has previously been published by our group [20]. Since expression of progenitor markers was previously detected in Defined medium and increased over time, we wanted to investigate if changes in culture media could enrich a certain cell phenotype. As expression of cardiac genes also increased, we wanted to study if it was possible to further favor cardiomyocytes with another culture medium. Therefore, the Defined medium was changed to Cardiac medium, including a pretreatment with 5-Azacytidine used for differentiation of cells, in 6-week-old HDS from 3 of the biopsies. These HDS were cultured in Cardiac medium for 3 additional weeks. As control HDS cultured in Defined medium for 9 weeks were used. To favor cells with endothelial phenotype, newly formed HDSs (1 week old) from 3 other biopsies were cultured for 3 weeks in Endothelial medium. HDS cultured in Defined medium for 4 weeks were used as control. To test the responsiveness of the HDS to stimulation of the Wnt/*β*-catenin signaling pathway, the GSK3 inhibitor SB216763 was added for a week to newly formed HDS from three additional biopsies. Due to the strictly limited access to human cardiac tissue, it was not possible to test all different conditions on cells from the same biopsy.

### 3.2. Morphology and Effects of Culture Media

From a cardiac biopsy, single cells were forced together by centrifugation to a flat pellet in the bottom of a tube. With time pellets rounded up and formed spherical structures that floated in the culture media. Those HDS were formed within a week and were made of a solid core that over time got slightly hollow after reorganization of the cells. After 9 weeks in culture HDS from two biopsies were dissociated, and proliferating monolayer cultures were established in both cases. Although no further studies on apoptosis or necrosis were conducted in this study, this shows that even after long time in HDS cells are viable and able to proliferate (data not shown).

**Figure 2:**
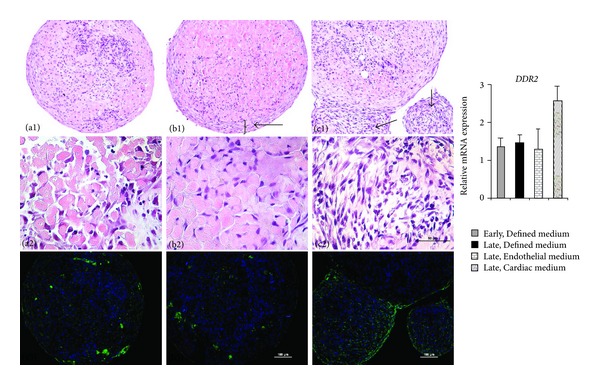
Morphology of HDS and effects of culture media. Histology of the HDS stained for HE, row 1 and 2. HDS cultured in (a) Defined medium at 6 weeks, (b) Cardiac medium at 9 weeks (Defined medium for 6 weeks followed by Cardiac medium for 3 weeks), and (c) Endothelial medium at 4 weeks (Defined medium for 1 week followed by Endothelial medium for 3 weeks) are shown. Arrows are showing the additional multilayer area (b1) and buds (c1). Row 3 shows staining with antibody against Ki67 (green) detecting proliferating cells. DAPI (blue) was used to counterstain nuclei. The histogram is showing relative mRNA expression of the cardiac specific fibroblast marker *DDR2*.

Different media were found to affect the cell proliferation as well as cell composition within the HDS (Figure 2). The morphology of the cardiomyocytes was best preserved after culture in Cardiac medium. Moreover, culture in this medium resulted in a thick multilayer of cells organized in sheets around the HDS. The additional multilayer area appeared different in the HDS cultured in Endothelial medium. In this medium, a more aggressive proliferation of cells was giving rise to buds, that is, new small spheres that were growing and with time shed of from the original HDS. In the buds, no cardiomyocytes were seen. 

Immunohistochemical staining for the proliferation marker Ki67 (Figure 2, row 3) showed positive cells through the culture time also in the serum-free Defined medium. When cultured in Cardiac or Endothelial media, which both contained serum, Ki67 expression was mainly located in the multilayer area on the surface of the HDS and in the buds. Due to fast proliferation, fibroblast overgrowth is a common issue in monolayer cultures [13]. To investigate if there was a fibroblast overgrowth, the gene expression of the previously described cardiac fibroblast marker *DDR2* [22] was measured in the HDS by qPCR. When cultured in Defined medium, HDS had a stable expression of *DDR2* over time. In Endothelial medium, with 5% serum, the *DDR2* expression was not increased compared to the Defined medium. A slight but not significant increase in expression level was noted in Cardiac medium, containing 2% serum (Figure 1).

**Figure 3:**
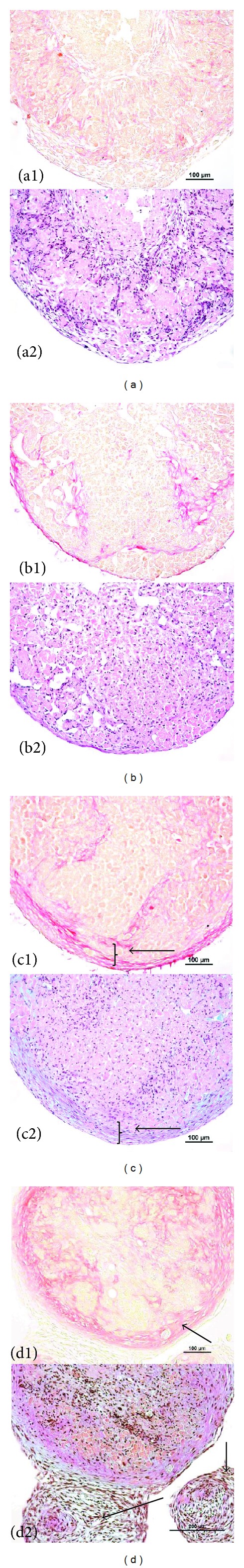
Extracellular matrix production. Histology of the HDS stained with Picro Sirius Red (red) in row 1 detecting collagen and AbvG (blue) in row 2 detecting proteoglycans. (a) HDS in Defined medium at 1 week. (b) HDS in Defined medium at 6 weeks. (c) HDS in Cardiac medium at 9 weeks (Defined medium for 6 weeks followed by Cardiac medium for 3 weeks) and (d) in Endothelial medium at 4 weeks (Defined medium for 1 week followed by Endothelial medium for 3 weeks). The multilayer area and the buds are marked by arrows.

Since the fibroblasts did not appear to proliferate to a large extent, as judged by histology and gene expression data, we then hypothesized that the role of fibroblasts in HDS could be ECM production rather than proliferation. In general, ECM production in 3D culture is considered as a sign of differentiation and functionality of the cells *in vitro* [23]. In the heart, cardiac fibroblasts are the cells responsible for producing most of the components of ECM [22]. Collagen type I predominates in the human heart, and with age these fibers increase in number and thickness [24]. In HDS cultured in Cardiac or Endothelial media, ECM was detected with staining for proteoglycans by AbvG and collagens by Picro Sirius Red (Figure 3). The multilayer areas contained large amounts of collagen. Only in these areas proteoglycans were detected with ABvG staining. In Defined medium, neither collagen nor proteoglycans was seen at 1 week. Over time collagen was produced but no proteoglycans were detected. Thus, Cardiac and Endothelial media gave a more sophisticated ECM, showing more tissue-like constructs.

### 3.3. Endothelial Phenotype

**Figure 4:**
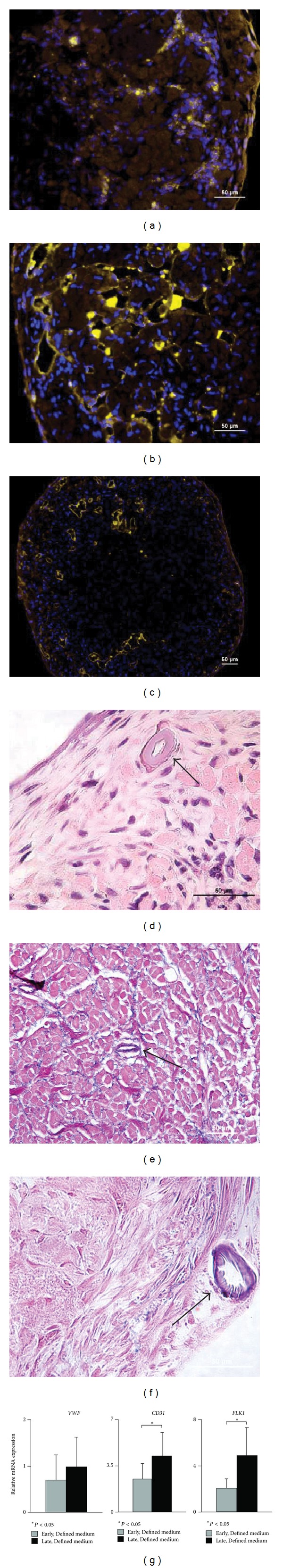
Endothelial phenotype in HDS. (a) Staining with antibody against *VWF* (yellow) shows randomly organized cells at 1 week in Defined medium. (b) At 6 weeks and (c) at 9 weeks the *VWF* positive cells were found mostly on the inside of cavities. DAPI (blue) was used to counterstain nuclei. (d) A vessel-like formation found in Endothelial medium at 4 weeks (Defined medium for 1 week followed by Endothelial medium for 3 weeks) in HE staining. (e) Millers elastin staining in human cardiac tissue (control) with blue staining for elastin in small vessels and (f) Millers elastin staining detecting another tubular structure in HDS cultured in Cardiac medium at 9 weeks (Defined medium for 6 weeks followed by Cardiac medium for 3 weeks). (g) Relative mRNA expression of endothelial-specific genes in HDS cultured in Defined media (*n* = 6) for 1 week (early) and 4–6 weeks (late).

Since no sign of fibroblast overgrowth was seen, the question remained which other cell types that could also proliferate in HDS. The first candidate for proliferation would be the endothelial cells since they are easy to culture. Immunohistochemistry performed on newly formed HDS after 1 week showed many cells positive for the endothelial marker *VWF*. Positive cells were organized together between the clusters of cardiomyocytes (Figures 4(a)–4(c)). After 6 weeks in culture, *VWF* positive cells were organized specifically on the inside of the cavities that appeared over time in culture. This intraluminal position remained also after 9 weeks. The phenomenon was observed in HDS cultured in Defined medium without addition of endothelial stimulating factors. Since intraluminal position is a natural location of endothelial cells *in vivo* this indicates that the endothelial cell phenotype is preserved and active within the HDS. 

In five of six biopsies analyzed with HE staining, vessel-like formations were found in all three media tested, but only from later time points. There is a possibility that those structures simply could be the rest products of an ineffective dissociation process, but since there were no similar structures seen at the early time points in any case, it is highly likely those were formed during the culture time. Miller's elastin staining on those sections showed structures similar to small vessels stained for elastin (Figures 4(d)–4(f)). Furthermore, those structures were followed in a number of serial sections. This showed formation of tubular constructs, suggesting an early vasculogenesis in HDS. In cardiac biopsies from the same location our group has previously isolated a small population of cells which was characterized as a population of endothelial progenitor cells [20]. Interestingly, these cells have been reported to be important for vasculogenesis [25]. Although not fully investigated in this study, the finding is highly interesting since vasculogenesis is an important event in the repair process after myocardial infarction. 

On the gene level, *CD31* and *FLK1* expressed by both mature endothelial cells and endothelial progenitor cells, increased significantly over time in Defined medium. *VWF* expressed by more mature endothelial cells increased only slightly (Figure 4(g)). In Endothelial medium, however, expression of these genes was rather decreasing compared to Defined medium (data not shown). This somewhat surprising finding might be due to the relatively high serum content of the Endothelial medium, which may favor proliferation at the cost of differentiation.

### 3.4. Cardiomyocyte Phenotype

**Figure 5:**
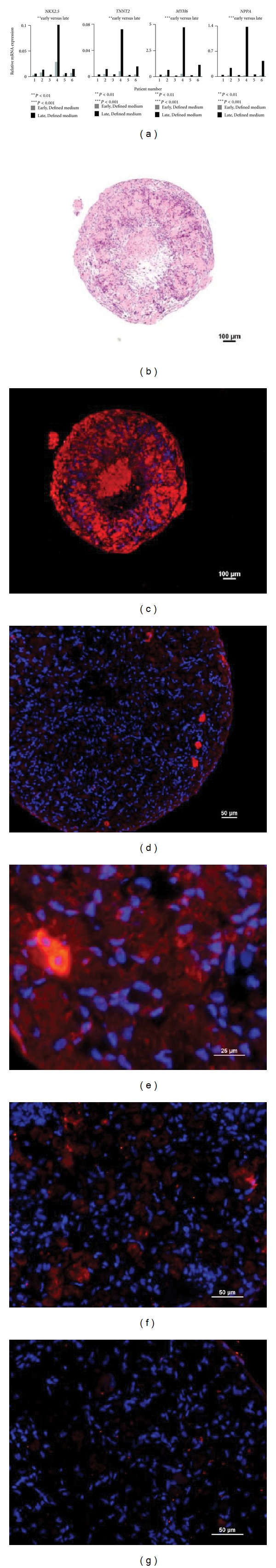
Cardiomyocyte phenotype in HDS. (a) Relative mRNA expression of four cardiac specific genes in early (1 week) and late (4–6 weeks) HDS cultured in Defined medium. (b) Histology of the HDS stained for HE at 1 week. (c) Staining with antibody against *α*-sarcomeric actin (red) expressed by cardiomyocytes at 1 week and (d) at 9 weeks. A larger magnification is shown to better visualize the staining. (e) Two cardiomyocytes keeping their intense expression of *α*-sarcomeric actin at 6 weeks. (f) Staining with antibody against TroponinT2 (red) at 1 week and (g) at 6 weeks. DAPI (blue) was used to counterstain nuclei.

Besides from endothelial cells, there are other candidates for proliferation in HDS. There is a hypothesis that dedifferentiated cardiomyocytes are able to reenter the cell cycle and start to proliferate [4, 5]. In our study, this theory could be partly supported by a significant increase in expression levels of the cardiac-specific genes *NKX2.5*, *TNNT2*, *MYH6,* and *NPPA* over time, although gene expression data only is not sufficient evidence for this event. Even though a notable biological variation between the patients was observed, the cardiac genes analyzed showed the same expression pattern over time (Figure 5(a)). When cultured in Cardiac medium, on the other hand, no increase in expression of cardiac-specific genes was noted (data not shown). This is in parallel to the behavior of the endothelial genes in HDS cultured in Endothelial medium. 

Within the HDS, cardiomyocytes were easy to distinguish because of their size and morphology. These cells were also strongly stained for *α*-sarcomeric actin in the newly formed HDS cultured in Defined medium (Figure 5). With culture time, however, the expression decreased. After 9 weeks, only few cardiomyocytes still expressed *α*-sarcomeric actin with the same intensity, although most cardiomyocytes were still positive. Cardiac troponin T protein expression was also showing this pattern even though a lower number of cells expressed this marker. Disorganization of the sarcomeres and dedifferentiation of cardiomyocytes illustrated by the declining protein expression of *α*-sarcomeric actin is a programmed cell survival mechanism [26] which is also needed to enable proliferation [5]. The simultaneous increase in expression of the cardiac-specific genes suggests that cardiomyocytes are active in long time cultured HDS. On the other hand this could also reflect different stages of cardiomyocyte maturity.

### 3.5. Expression of Progenitor Cell Markers and Early Cardiac-Related Markers

Besides from dedifferentiation of existing cardiomyocytes, another possibility is an ongoing differentiation of progenitor cells within the HDS to new cardiomyocytes that start expressing *α*-sarcomeric actin. This could be an explanation of the few *α*-sarcomeric actin positive cells with high staining intensity at the late time points. The possibility for this option should be improved by the diversity within the paracrine communication since the progenitor cells are cocultured with adult cardiomyocytes in HDS, and the paracrine effects are encouraged by the 3D environment. The positive effects of such self-conditioned medium have been described and used by others [5].

**Figure 6:**
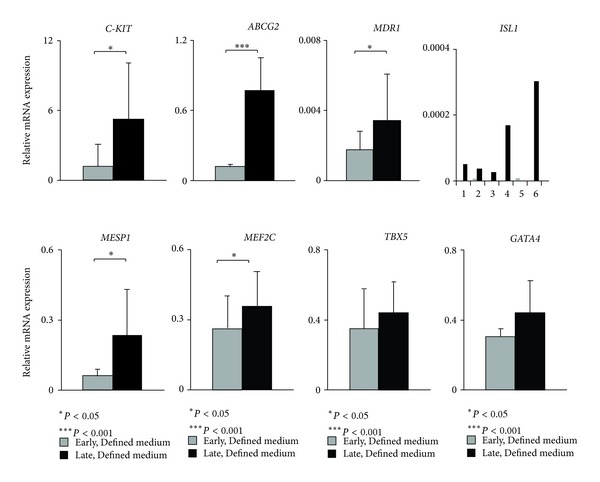
Relative mRNA expression of markers for progenitor cells and early cardiac-related markers. Comparison between early time point when HDS were formed and late after 4–6 weeks in Defined medium (*n* = 6).

Expression of cardiac progenitor genes was analyzed by qPCR in HDS cultured in Defined medium. Expression was compared for an early (1 week) and a late (4–6 weeks) time point in HDS (Figure 6). The progenitor-related genes *C-KIT*, *ABCG2, *and *MDR1* were significantly upregulated over time. *ISL1*, a fetal CPC marker, was detected in 2 of 6 patients tested in the newly formed HDS. With culture time, its expression was upregulated in 5 of 6 patients. *ISL1+* cells are known to be able to differentiate into cardiac, smooth muscle, and endothelial cells [27–29]. After birth, the number of *ISL1+* cells is extremely low [29]. Detection of *ISL1* gene expression in our material, with an average age of the donors of 71 years, indicates that HDSs provide a good environment for preservation of the stem-cell phenotype. A significantly increased expression was seen for the early mesodermal marker *MESP1* over time. The early cardiac-specific transcription factor *MEF2C *also had a significant higher expression at the later time point, while no significant increase was seen for the other early cardiac transcription factors *TBX5* and *GATA4*. *MEF2C* has previously been described as a direct target of *GATA4* and Isl1 [30]. Transfection with the three early cardiac specific transcription factors *GATA4*, *MEF2C *and *TBX5* could transform mouse fibroblasts into functional cardiomyocytes [31], pointing out their importance for cardiomyocyte differentiation. 

**Figure 7:**
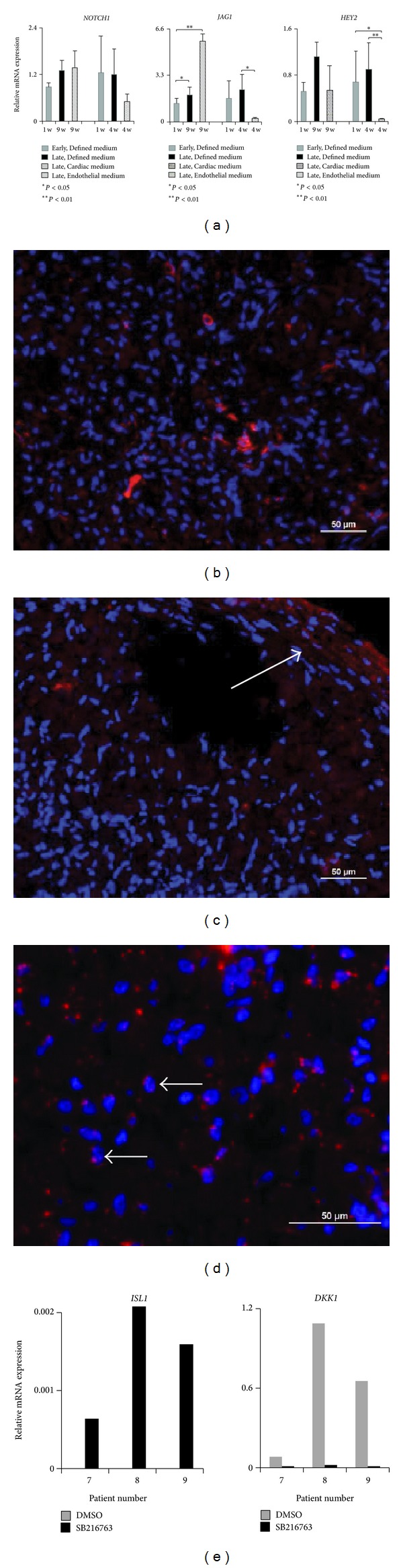
Activities within the progenitor cell regulating Notch and Wnt pathways. (a) Relative mRNA expression of the receptor *NOTCH1, *its ligand *JAG1,* and downstream gene *HEY2. *Early and late time points of HDS cultured in Defined media were compared either to HDS cultured in Cardiac (*n* = 3) or Endothelial (*n* = 3) media. (b) Staining with antibody against *NOTCH1* (red) at 6 weeks in HDS cultured in Defined medium. (c) When cultured in Cardiac medium, *NOTCH1* staining was mostly located to the additional multilayer area (arrow) at 9 weeks. (d) Active form of *β*-catenin (red) was detected in many cells in the HDS, illustrated by the nuclear position of the staining (arrows). DAPI (blue) was used to counterstain nuclei. (e) Relative mRNA expression of *ISL1* and *DKK1* after one week of stimulation with GSK inhibitor SB216763, DMSO as negative control (*n* = 3).

In addition to expression of cardiac progenitor markers within the HDS, activity in the progenitor cell-associated NOTCH pathway was also detected (Figure 7). *NOTCH1* activity has been shown to be required for differentiation of CPC cells [10]. In the HDS culture, *NOTCH1* protein expression was detected by immunohistochemical staining throughout culture time. In Defined medium, *NOTCH1* positive cells increased from 1 to 6 weeks, although there was no significant increase of *NOTCH1* expression on the gene level. When HDS was cultured in Cardiac medium, *NOTCH1* protein was found to be specifically located to the additional multilayer region in the HDS, colocalized to the ECM. JAG1, a ligand to the NOTCH receptor, was not detected by immunohistochemistry (data not shown). On the gene level, on the other hand, the expression of *JAG1* increased significantly when cultured in Cardiac medium compared to Defined medium. The dramatic increase of *JAG1* in this medium indicates the possibility of induction of differentiation of CPC. The opposite, a significant downregulation of *JAG1* expression, was seen in HDS cultured in Endothelial medium compared to HDS in Defined medium. Gene expression of the helix-loop-helix transcriptional repressor* HEY2*, which has been reported to act downstream *NOTCH1* to affect Gata depending genes [32], was also analyzed. Like *JAG1, HEY2* expression decreased significantly in Endothelial medium compared to Defined medium.

In addition to Notch, activity in the Wnt/*β*-catenin signaling pathway was also studied in newly formed HDS cultured in Defined medium. Immunohistochemistry was performed with an antibody specific against the dephosphorylated, active form of *β*-catenin, which has the capacity to enter the nucleus and start transcription. This active form of *β*-catenin was detected in many cells in the HDS. The activity is further illustrated by the nuclear position of the staining in Figure 7(d). *β*-catenin signaling has been reported to be required for Isl1 expression [9]. The canonical Wnt/*β*-catenin pathway can promote expansion of *ISL1+* progenitors through addition of a GSK3 inhibitor which dephosphorylates *β*-catenin [8, 28]. Since this active form of *β*-catenin was detected on protein level in HDS, we hypothesized that the HDS could be used as a model for studying the interactions between Wnt and Isl1. To test this, a GSK3 inhibitor was added to newly formed HDS for one week. This resulted in expression of *ISL1 *compared to DMSO control where no expression was detected. In addition, downregulation of the Wnt inhibitor *DKK1 *was noted (Figure 7(e)). These findings support the suitability of HDS for studies of the role of GSK3 inhibitors in cardiac research. This is of particular interest since GSK3 inhibitors have become interesting pharmaceutical candidates, for example, in diabetes and Alzheimer's disease [33], and, lately, attention has also been paid to the role of GSK3 in heart disease [34].

### 3.6. Benefits and Limitations Using HDS

While embryonic and fetal material provides an important platform for development studies, clinical trials are directed towards elderly individuals. Relatively high age of the subjects included in this study gives perhaps a more realistic view of the cells and their expressed markers within a common patient group. Compared to most other culture systems including cardiac cells and in particular CPC, the HDS model allows for the presence of cardiomyocytes which are important both for a paracrine signaling and for studies within the regenerative medicine field. Although advantageous in this case, the use of adult material also has limitations. We assume that the age affects the differentiation potential of the cells. Moreover, the biological variation between the patients is also a factor to consider when working with primary isolated cells. Also there were not enough cells for methods like FACS sorting or western blotting. 

## 4. Conclusion

3D environment and high cell density in HDS play an important role in restoring and supporting the phenotypes of cells represented in cardiac tissue including the cardiomyocytes. In the HDS culture, these features are advantaged and enable studies of adult human cells. By using different culture media, it was possible to affect the cells within the HDS in different ways. Defined media gave significant increase in both cardiac- and progenitor-specific markers and also an intraluminal position of endothelial cells. Cardiac media showed indication of differentiation and maturity of HDS considering the ECM production and activities within Notch regulation but no additional cardiac differentiation. Endothelial media gave no positive effects on endothelial phenotype but increased proliferation without fibroblast overgrowth. Moreover, it was possible to affect the Wnt signaling in HDS by addition of a GSK3 inhibitor. Taken together, these findings show the suitability of HDS as an *in vitro *model for studies of cardiomyocyte-, endothelial-, and stem-cell biology.
